# The Effects of Motor Imagery on Static and Dynamic Balance and on the Fear of Re-Injury in Professional Football Players with Grade II Ankle Sprains

**DOI:** 10.3390/healthcare12141432

**Published:** 2024-07-17

**Authors:** George Plakoutsis, Elias Tsepis, Konstantinos Fousekis, Eleftherios Paraskevopoulos, Maria Papandreou

**Affiliations:** 1 Laboratory of Advanced Physiotherapy, Department of Physiotherapy, University of West Attica, 12243 Athens, Greece; 2 Department of Physiotherapy, University of Patras, 26504 Patra, Greece

**Keywords:** motor imagery, football, ankle sprain, balance, fear of re-injury

## Abstract

Lateral ankle sprains are one of the most frequent athletic injuries in football, causing deficits in balance. Motor Imagery (MI) has been successively included in sports rehabilitation as a complementary therapeutic intervention. The aim of the present study was to explore the effects of MI on static and dynamic balance and on the fear of re-injury in professional football players with Grade II ankle sprains. Fifty-eight participants were randomly allocated into two groups: First—MI group (*n* = 29) and second—Placebo group (*n* = 29), and they each received six intervention sessions. The first MI group received MI guidance in addition to the balance training program, while the second Placebo group received only relaxation guidance. One-way ANOVA showed statistically significant results for all variables, both before and 4 weeks after the interventions for both groups. The *t*-test showed statistically significant differences between the two groups for static balance for the right lower extremity (t = 3.25, S (two-tailed) = 0.002, *p* < 0.05) and also for heart rate (final value) in all time phases. Further research is needed in order to establish MI interventions in sports trauma recovery using stronger MI treatments in combination with psychophysiological factors associated with sports rehabilitation.

## 1. Introduction

Football (soccer) is one of the most famous sports around the globe with a constantly increasing rate of participants [1,2,3]. The Federation Internationale de Football Association (FIFA) reports that approximately 270 million football players are registered globally [2]. The Brazilian Football Confederation reports that there are 2.1 million professional athletes and 11.2 million amateur athletes without taking into account recreational athletes in Brazil [2,4]. Football is a multiplayer team sport [1] that requires physical skills in order to confront the high levels of exertion during a match [2]. It is a sport demanding high physical activity, involving aspects such as physical contact, acceleration, deceleration, jumps, pivots, changes of direction, and physical, physiological, technical, and tactical skills [1,2]. Therefore, football players are at a high risk of injuries (e.g., ankle sprains) at both professional and amateur levels, affecting the success of their teams and the level of participation of the injured athlete [2,5].

Acute lateral ankle sprains (LAS) are one of the most common musculoskeletal injuries in sports, accounting for up to 40% of all sports-related injuries [6,7]. Most ankle injuries occur in athletic activities that require jumping and landing techniques such as in football [8], while the majority of acute ankle sprains in football occur during the first and the last 15 min of the game [2]. LAS can be categorized based on their severity [9]: Grade I involves a mild stretching of the ligaments (anterior talofibular ligament—ATFL and calcaneofibular ligament—CFL) without microscopic rupture or joint instability [9,10]. Grade II involves a moderate partial ligament rupture of the ATFL and stretching of the CFL, along with pain, swelling, functional limitation, and slight instability [9,10]. Grade III is a severe rupture of the ATFL and CFL and also involves the stretching of the posterior talofibular ligament (PTFL) along with pain, swelling, hematoma, functional impairment, and instability [9,10]. An estimated 2 million ankle sprains occur annually in the United States, resulting in healthcare costs of $2 billion [6]. In the United Kingdom, ankle sprains were the most prevalent injuries in the emergency room, while in the Netherlands, €187.2 million is spent on treating acute ankle sprains, thus affecting sports training and sports performance in football athletes [11].

However, acute ankle sprains can cause a variety of physiological deficits, including muscle weakness, lowered muscle-reaction time, limited range of motion (ROM), and limited proprioception and balance, all of which affect static and dynamic postural control [7,12,13,14,15]. Sports experts usually perform postural control assessments in order to evaluate deficits in balance after an acute ankle sprain or to evaluate the progress of the rehabilitation intervention [13].

Static balance is defined as the ability to create a stable base of support and to maintain this position while minimizing movement of the body and extremities [13,16,17]. Static balance assessment can be performed objectively via instruments such as force platforms [13,16,18]. Static balance assessment using the Center of Pressure (CoP) gives the opportunity to evaluate altered parameters after an acute ankle sprain in footballers, such as postural sway while in a single limb stance (Single-Leg Stance Test—SLST) [16,19]. On the contrary, dynamic balance is defined as the ability to maintain a base of support while performing a movement (e.g., movement of the lower extremity while maintaining the base of support) [13,20]. Dynamic balance has also been associated with injuries in the lower extremities [20] and its dynamic measures (e.g., Y Balance Test-YBT) are more closely mimicking sports demands in comparison with static balance assessment [13,21].

However, psychological responses appear to play an important role after an acute LAS, affecting the healing process [22] and the return to play period [23]. These responses include anxiety, depression, decreased self-confidence, and especially fear of re-injury [24]. Athletes who are physically but not psychologically prepared to return to play might have lower performance levels due to the increased levels of fear of re-injury [23,25]. Motor Imagery (MI) has been progressively included in sports rehabilitation as an adjunct therapeutic modality either for injury management or for improving sports performance practices (e.g., self-efficacy, injury anxiety, and fear of re-injury) [26]. In fact, MI has become one of the most popular therapeutic techniques among athletes for performance optimization purposes [26,27] in training and during competitions, but the use of MI during sports rehabilitation has received limited research attention [26,27,28,29]. MI can also reproduce responses of the autonomic nervous system (ANS), which usually take place during physical exercise (e.g., football, physical recovery). On the peripheral nervous system, these responses include increased heart rate (HR) and blood oxygen saturation rate (SPO_2_) [26]. Therefore, the ability to use MI and its effectiveness can be assessed with the evaluation of the ANS responses or with reliable and valid psychometric tools such as the VMIQ-2-GR [30]. MI is defined as a dynamic cognitive process of mental representation of movements without its actual execution [26,31,32,33]. These images are commonly visualized via a state of relaxation and a specific outcome in mind (e.g., fear of re-injury) [34].

While the effectiveness of MI in sports performance is well-established, there is an insufficient amount of research focusing on injury rehabilitation after an acute LAS [26,28,29,35] and its effect on athletes’ return to play criteria. The intervention of psychological strategies such as MI during the rehabilitation after an acute LAS in a professional football player may also reduce the levels of fear of re-injury and, therefore, accelerate the time of return to play. It seems reasonable to explore the effect of MI on both static and dynamic balance in professional football players after an acute LAS and its relationship to decreasing the fear of re-injury. Therefore, it is extremely important for all clinical sports therapists to establish an effective therapeutic modality that takes into account psychological factors both in terms of acute LAS rehabilitation and return to play. The aim of the present study was to investigate the effects of MI on balance and on the fear of re-injury during the pre-discharged stage of rehabilitation in professional football players who have experienced a Grade II ankle sprain.

## 2. Materials and Methods

### 2.1. Participants

A total of 66 professional football players with a LAS were initially recruited by the stratified random sampling method. They were evaluated by a sports medicine doctor in a private clinic with the use of ultrasound and objective clinical tests. Three of them were identified with functional ankle instability and therefore excluded from the study, and five left the procedure. Ultimately, 58 athletes aged 18–35 (mean age = 20.8 ± 3.2) met the inclusion criteria and agreed to participate in the study. All of them had a Grade II LAS either in the left or right leg. All athletes were professional football players from the Greek national levels of Super League 1, Super League 2, and Football League. Athletes were recruited from the Laboratory of Advanced Physiotherapy of the University of West Attica and volunteered to participate in this study. All professional athletes were informed that they could withdraw from the study at any time without any consequences. All athletes were informed about the procedures involved in the study and signed a written informed consent. The study was accepted by the Ethics Committee of the University of West Attica (No. 18030), and it was registered in TCTR clinical trials (No. TCTR20210720004). The study protocols were in accordance with the Declaration of Helsinki.

Athletes were randomly divided into 2 groups using the method of closed envelopes. The first MI group consisted of 29 professional football players (50%), and the second Placebo group also consisted of 29 professional football players (50%). The age of participation in both groups was 18–35 years (MI group: mean age 20.5 ± 3.3, Placebo group: 21.2 ± 3.1). The anthropometric characteristics of the athletes were as follows: mean height was (1.76 ± 0.06), mean weight was (69.6 ± 7.8), mean BMI was (22.3 ± 1.9), mean years of sport participation (11.1 ± 2.7) and mean training hours per week (12.1 ± 1.5). Forty-six athletes were dominant in the right leg, whereas 12 were dominant in the left leg. Thirty-nine athletes had a right leg LAS, whereas 19 had a left leg LAS. Thirty-eight athletes had a previous right leg LAS, 12 athletes had previous left LAS, and 8 athletes had both right and left previous LAS. Thirty athletes had a history of 1 previous LAS, 22 athletes had a history of 2 previous LAS, and 7 athletes had a history of 3 previous LAS.

The inclusion criteria of the study were (a) professional football players aged 18–35, (b) participating in Greek National levels, Super League 1, Super League 2, and Football League, (c) active athletic training in football >5 years (professional level), (d) >10 h of training per week, (e) suffered from a LAS and currently in the pre-discharged stage of rehabilitation (return to play period), (f) have successfully completed previous stages of rehabilitation, and (g) have been diagnosed with a Grade II LAS from a sports medicine doctor with at least 5 years of experience in the sports field through ultrasound and clinical tests. The exclusion criteria of the study were: (a) visual, vestibular, and neurological impairments, (b) a history of fracture, sprain, or musculotendinous injury of the lower limbs during the last 6 months, (c) presence of functional ankle instability with the use of the Greek version of the Identification of Functional Ankle Instability questionnaire (IdFAI-GR) [36] (d) surgical operation of the lower limbs during the last year, and (e) a history of concussion during the last 6 months.

### 2.2. Procedures

This study performed an experimental design and compared the MI group with the Placebo group. The testing procedures took place in 2 phases before and after the MI interventions, and the total duration of the protocol lasted 4 weeks. Prior to testing in Phase 1, athletes were made familiar with the Single Leg Stance Test (SLST) and the Y Balance Test (YBT). An experienced sports physiotherapist with 5 years of experience in the sports medicine field briefed them 1 week in advance about the execution of the optimal technique through a live demonstration. Their body mass was measured to the nearest 0.1 kg using the electronic scale Mi Body Composition Scale 2 (Xiaomi Inc., Beijing, China), and their height was measured to the nearest 1 cm through a portable stadiometer (SECA Instruments Ltd., Hamburg, Germany) [37]. The SLST was performed on a reliable and valid portable force platform, the KForce Plates system (Kinvent, Montpellier, France) [16,37] and the parameters were recorded on a Samsung tablet (Samsung Electronics Co., Ltd., Suwon, Republic of Korea) in order to evaluate the Center of Pressure (CoP) and consequently the postural sway. The SLST was performed in 2 conditions, both in open and closed-eye conditions. The YBT was performed using the reliable Y Balance Test Kit^TM^ in order to assess dynamic balance [21]. Upon completion of both the SLST and YBT testing procedures, athletes had to complete the Causes of Re-injury Worry Questionnaire—CR-IWQ, a reliable and valid instrument, in order to evaluate the levels of fear of re-injury [25] before the intervention protocol. The measurements were carried out at each team’s own sports facilities in order to ensure that they would not be psychologically affected by a different testing environment. Phase 2 took place upon completion of the intervention protocol exactly 4 weeks after Phase 1. The timeframe was determined based on the duration of the pre-discharged stage of rehabilitation in order to evaluate the variables of balance and fear of re-injury during the return to play period.

### 2.3. Main Outcome Measures

#### 2.3.1. Single Leg Stance Test

**Figure 1:**
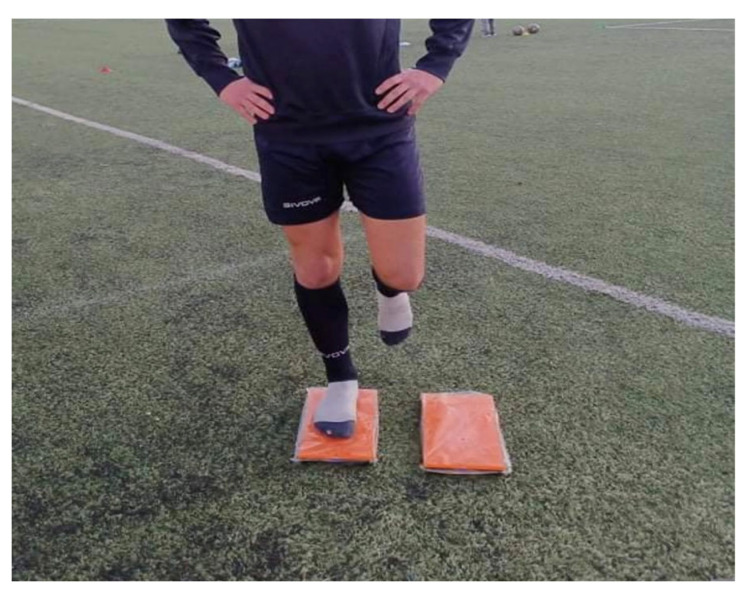
Single Leg Stance Test with the use of KForce plates.

Athletes were instructed to remove their shoes and to step on the previously calibrated (according to the manufacturer’s guidelines) KForce Plates system during both Phases (1 and 2). They then had to stand in an upright position with a straight torso, initially with the right leg and then with the left. During the open-eye (OE) condition, they had to remain motionless, keep their eyes open, place their hands on their hips, flex their non-weight-bearing leg slightly at the knee and hip joint, and place and keep the weight-bearing foot in a straight position [16,18]. The procedure started after the initial sound notification of the KForce Plates system application and lasted 20 s for each leg with a break of 10 s. The assessment was repeated if the athlete hopped on the weight-bearing leg, touched the ground with the non-weight-bearing leg, or removed his hands from the hips. Then, the same timeframe was followed for the left leg. The exact same procedure was executed in the closed eye (CE) condition. The KForce Plates system (Kinvent) is a portable force platform that sends biofeedback (audio and visual) to a smartphone or tablet via the KForce application. The dimensions per plate are 30 mm × 320 mm × 160 mm, the max weight is 300 kg per plate with an accuracy of 500 g, the radio range is up to 20 m, the transmission frequency is 2.4 GHz band, the acquisition frequency is up to 75 Hz and it weighs 1.6 kg. We used the default settings of the KForce plates for the testing procedure in order to measure SLST (Center of Pressure—CoP), and we exported raw data for analysis with the use of the KForce application. The KForce Plates system has been proven reliable with ICC = 0.87 for measuring SLST [16] and ICC = 0.99 for measuring Countermovement Jump [37], Figure 1.

#### 2.3.2. Y Balance Test

Athletes were instructed to step on the YBT Kit after they had removed their athletic shoes. The assessments of the YBT took place during both Phases (1 and 2), and athletes had to stand on their right leg first on the central footplate with their toes placed just before the starting line. Athletes were instructed to maintain a single-leg stance, to keep their hands on their hips, and with their free limb, reach as far as possible to the anterior, the posteromedial, and the posterolateral directions by pushing the reach indicator. All participants were instructed to perform 3 trials in each direction in relation to the stance limb. The maximal reach distance was measured using a tape measure at the point where the most distal part of the foot was able to reach. The assessment was repeated if the athlete touched the ground with the free limb, failed to maintain reach contact with the reach indicator, used the reach indicator for support in order to maintain balance, or failed to return the free limb to the initial position. Limb length was measured by the investigator with the use of a tape measure after the athlete completed the testing procedure. Limb length measurements were obtained in centimeters, measuring both of the athlete’s limbs in the supine position from the anterior superior iliac spine to the distal aspect of the medial malleolus [21,38,39]. YBT reach distances were normalized using limb length in the following equation: reach distance/limb length × 100. The composite reach score was calculated using the following equation: mean anterior + mean posteromedial + mean posterolateral/limb length × 3 × 100. The YBT has shown good reliability with ICC (0.85–0.91) [21].

**Figure 2:**
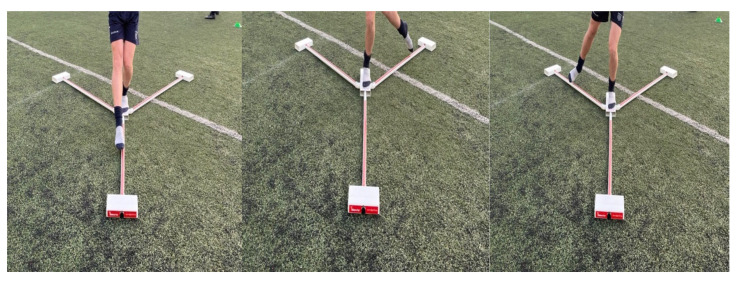
Y Balance Test.

The Y Balance Test Kit is composed of a stance platform and an attachment of 3 pipes with different directions (anterior, posteromedial and posterolateral). The anterior pipe is placed at an angle of 45° with the posterior pipes, which are placed at an angle of 135°. Each pipe encompasses a measurement with an accuracy of 5 mm increments [21], Figure 2.

#### 2.3.3. Causes of Re-Injury Worry Questionnaire (CR-IWQ)—Fear of Re-Injury

Athletes were informed about the completion of the Causes of Re-injury Worry questionnaire (CR-IWQ). The CR-IWQ was delivered to the athletes upon completion of the balance testing (static and dynamic) in both Phases (1 and 2). The duration of the completion of the CR-IWQ was approximately 5 min. The CR-IWQ is a questionnaire with twelve items and 2 perspectives (re-injury worry due to rehabilitation and re-injury worry due to the opponent’s ability). Athletes have to answer the twelve items spontaneously, of which 1, 2, 3, 5, 7, 8, 9, and 12 concern the re-injury worry due to rehabilitation, and items 4, 6, 10, and 11 concern the re-injury worry due to the opponent’s ability. Athletes have to rate each item on a 7-point Likert scale where 1 = never, 4 = moderately, and 7 = very much. The CR-IWQ has shown high reliability in both perspectives: (a) Re-injury worry due to rehabilitation, ICC = 0.94, score range 8–56 (the higher the score, the greater the athlete’s re-injury concern), (b) re-injury worry due to the opponent’s ability, ICC = 0.98, score range 4–28 (the higher the score, the greater the athlete’s re-injury concern) [25].

#### 2.3.4. Vividness of Movement Imagery Questionnaire-2

The VMIQ-2 is a psychometric tool with twelve questions and 3 perspectives [External Visual Imagery—(EVI), Internal Visual Imagery—(IVI), and Kinesthetic Visual Imagery—(KVI)]. Athletes have to visualize themselves performing twelve motor imagery actions from three different imagery aspects: [EVI (3rd person perspective), IVI (1st person perspective), and KVI (feeling the movement)]. Also, they have to rate the vividness on a 5-point Likert scale from 1 (perfectly clear and vivid) to 5 (no image at all) [30]. The VMIQ-2 twelve motor imagery actions are as follows: 1. Walking, 2. Running, 3. Kicking a stone, 4. Bending down to pick up a coin, 5. Running upstairs, 6. Jumping sideways, 7. Throwing a stone into water, 8. Kicking a ball in the air, 9. Running downhill, 10. Riding a bike, 11. Swinging on a rope, 12. Jumping off a high wall. The VMIQ-2 scores can vary from high to low imagery ability (VMIQ-2 score > 36 and VMIQ-2 score < 26). The Greek version of the VMIQ-2-GR has been proven reliable and valid, and it showed acceptable factorial, concurrent, and construct validity (ICC > 0.92) [30].

#### 2.3.5. Intervention Protocol

**Figure 3:**
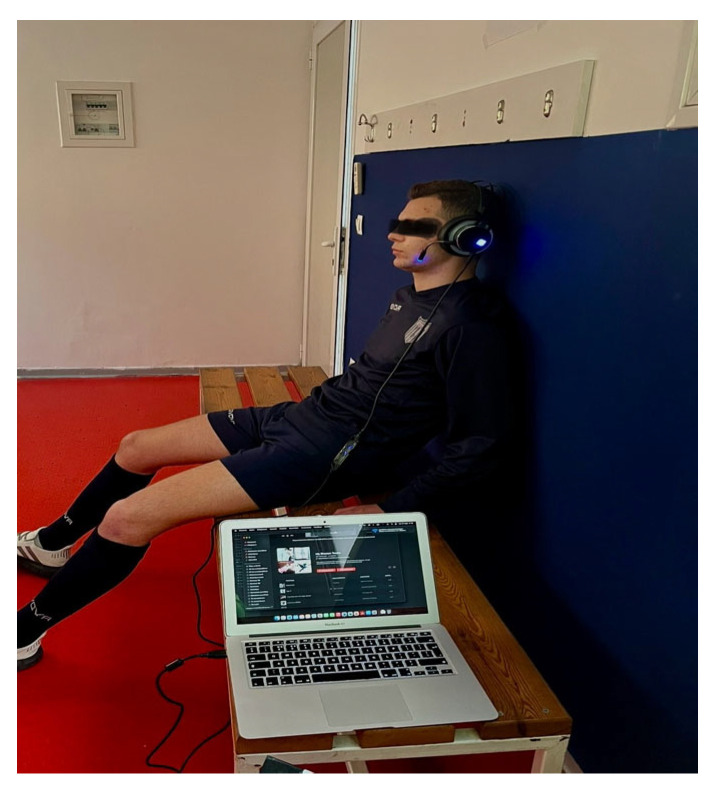
MI intervention.

Athletes in both groups (MI and Placebo) received a physiotherapy balance training program 2 days after the completion of Phase 1. The intervention protocol lasted 4 weeks (pre-discharged stage of rehabilitation) with a total of 6 sessions for each athlete. The balance training program included Part 1—an 8 min warm-up with the following exercises: (a) Running (60 s), (b) Jumping jacks (40 s), (c) Linear knee raise (10 reps), (d) Squat (10 reps) and (e) Leg swing (10 reps); Part 2—15 min of Balance exercises: (a) One-legged stance on an exercise mat 3 × 45 s hold, (b) Jump from one leg to the other and control landing for 4 s, 3 × 10 reps, (c) One-legged stance on a balance board 3 × 45 s hold, (d) One-legged stance on the Balance board with the knee flexed 3 × 10 knee flexions, (e) Two-legged squat on a Balance board 3 × 45 sec hold and Part 3. 7 min of lower limb static stretching exercises [19,40,41,42], Figure 3.

Upon completion of the balance training program athletes in the MI group received individually the MI intervention protocol in addition to the balance training program. At the beginning of each session, athletes in both groups completed the VMIQ-2-GR. The MI group and the Placebo group received six individual sessions of MI and Placebo MI in addition to their balance training program with a total duration of 50 min (20 min of MI and Placebo). Athletes were asked to sit in a comfortable and relaxed position in quiet and low-light environmental conditions. The instructions of the MI protocol were given by the researcher of the study and had been recorded in a professional recording studio by a sound engineer, thus minimizing interpretation bias. The researcher was certified in Graded Motor Imagery, and the final recordings were made with the use of WaveLab version 9 and Cubase version 10, along with a condenser microphone (NT1, 4th generation, Rode, Sydney, Australia). The reproduction of the instructions was made using the same computer (MacBook Pro, M1, Apple Inc., Cupertino, CA, USA) and the same headphones (Keiji, HD-2400G, Zeroground, Athens, Greece). During the sessions, a palm oximeter (Zacurate, 500C, Stafford, TX, USA) was placed on the index finger of each athlete in order to evaluate SPO_2_ and HR fluctuations during the intervention protocol in both the MI and Placebo groups in 5 different phases (phase 1 = initial value − 0 min, phase 2 = 5 min, phase 3 = 10 min, phase 4 = 15 min and phase 5 = 20 min). The means of phases 2, 3, 4, and 5 constituted the final value for the variables SPO_2_ and HR.

In the MI group, athletes initially received relaxation instructions followed by the same balance training program. Athletes had to mentally rehearse the sequence of the same balance training session, including the duration and the repetition of each exercise. They were also instructed to imagine themselves while performing the exercises and to feel their body and movements as vividly as possible. In the Placebo group, athletes received only relaxation instructions without any MI action.

#### 2.3.6. Statistical Analysis

As previous identical methodological designs have not been applied by a researcher in the field of MI, our sample size estimation was calculated based on a study of twenty young football players [43]. Measurement of dynamic balance through the YBT before and after the intervention allowed the investigators to continue with power analysis. The G*Power software (version. 3.1; Franz Faul, Kiel University, Kiel, Germany) was used in order to calculate the sample size with f = 0.25 and partial *η*^2^ = 0.06, considering the possible number of participants and that there will be a 4-week intervention program [44]. Therefore, with values (α) = 0.05, power (1 − β) = 0.95, with 2 equal groups of participants (first—MI intervention group, second—Placebo intervention group) and a total of 10 assessment procedures, the effect size ranges between medium and large. It was estimated that the minimum number of participants needed to start the research process was sixty-six athletes.

Descriptive statistics were used, with means and standard deviations for continuous variables, and count and proportion were reported for categorical variables. The chi-square trend test and the chi-square test were used in order to determine if there were statistically significant differences in the anthropometric characteristics of the athlete population [45], Appendix A. Normality check of the data was performed through the Kolmogorov–Smirnov test. Repeated measures analysis of variance (ANOVA) was performed in order to compare the means of variance within the subject effects ‘time’ factor after 4 weeks and between the 2 groups of effects [46]. *t*-tests for independent variables were used in order to determine statistically significant differences between the 2 groups [47]. The statistical significance was set at *p* < 0.05. To estimate clinical relevance and to quantify the differences between the 2 groups, partial eta squared (*η*^2^) was calculated. To evaluate the effect sizes, *η*^2^ was classified as *η*^2^ = 0.01, *η*^2^ = 0.06, *η*^2^ = 0.14, indicating small, medium, and large effect sizes, respectively [44,48]. All analysis was performed using the SPSS v. 26 statistical package (Statistical Package for the Social Sciences, SPSS Inc., Chicago, IL, USA).

## 3. Results

### 3.1. Static Balance—SLST

Repeated measures analysis ANOVA within subjects for testing static balance (SLST) through the portable KForce Plates before and after 4 weeks (Phases 1 and 2) showed statistically significant results in the OE condition for both legs (Left leg: F = 10.049, S = 0.002, *p* < 0.05, Right leg: F = 5.523, S = 0.022, *p* < 0.05), while no statistically significant differences were observed between the two groups for the left leg (Table 1).

The *t*-test for independent samples showed statistically significant differences between the two groups for the right leg in the OE condition (*t* = 3.25, S (two-tailed) = 0.002, *p* < 0.05). The means of the CoP in the OE condition for the right leg showed a statistically significant decrease of 47.1 mm^2^ in the first—MI group compared to the second—Placebo group, which showed a decrease of 42.8 mm^2^. The means of the CoP in the CE condition for both legs did not show statistically significant differences.

Partial variance effect size (*η*^2^) showed a large effect size (*η*^2^ = 0.9) and observed power = 0.637 (α = 0.05) for the right leg in the OE condition.

### 3.2. Dynamic Balance—YBT

Repeated measures analysis ANOVA within subjects for testing dynamic balance (YBT) before and after 4 weeks (Phases 1 and 2) and between the two groups showed statistically significant results in both legs (Left leg: F = 7.622, S = 0.008, *p* < 0.05, Right leg: F = 11.451, S = 0.001, *p* < 0.05), while no statistically significant differences were observed between the two groups in both legs (Table 2).

Partial variance effect size (*η*^2^) showed medium effect size (*η*^2^ = 0.12) and observed power = 0.774 (α = 0.05) for the left leg, while for the right leg, it showed large effect size (*η*^2^ = 0.17) and observed power = 0.914 (α = 0.05).

### 3.3. Causes of Re-Injury Worry Questionnaire (CR-IWQ)—Fear of Re-Injury

Repeated measures analysis ANOVA within subjects for testing the CR-IWQ before and after 4 weeks (Phases 1 and 2) and between the two groups showed statistically significant results regarding the levels of the fear of re-injury in both factors (a. re-injury worry due the rehabilitation: F = 13.488, S = 0.001, *p* < 0.05, b. re-injury worry due to the opponent’s ability: F = 4.737, S = 0.034, *p* < 0.05) while no statistically significant differences were observed between the two groups (Table 3). The mean score of the factor ‘re-injury worry due to the rehabilitation’ was decreased by 6.2 points in the 1st MI group compared to the 2nd Placebo group, which decreased by 1.3 points between phases (1 and 2). The mean score of the factor ‘re-injury worry due to the opponent’s ability’ was decreased by 2 points in the 1st MI group compared to the 2nd Placebo group, which increased by 0.8 points between phases (1 and 2).

Partial variance effect size (*η*^2^) showed a large effect size (*η*^2^ = 0.19) and observed power = 0.95 (α = 0.05) for factor (a), while for factor (b), it showed a medium effect size (*η*^2^ = 0.07) and observed power = 0.57 (α = 0.05).

### 3.4. Vividness of Movement Imagery Questionnaire—VMIQ-GR

Repeated measures analysis ANOVA within subjects for testing the VMIQ-2-GR after 4 weeks (6 sessions) between the two groups showed statistically significant results of the levels of the athletes’ ability of MI in all three factors (EVI: F = 13.697, S = 0.000, *p* < 0.05, IVI: F = 12.191, S = 0.000, *p* < 0.05, KVI: F = 6.996, S = 0.000, *p* < 0.05) while no statistically significant differences were observed between the two groups (Table 4). The mean score of the EVI factor showed a decrease of 7.4 points in the 1st MI group compared to the 2nd Placebo group, which showed a decrease of 5.8 points. The mean score of the IVI factor showed a decrease of 4.1 points in the first MI group compared to the second Placebo group, which showed a decrease of 6 points. The mean score of the KVI factor showed a decrease of 6.5 points in the first MI group compared to the second Placebo group, which showed a decrease of 2.3 points between the phases (1 and 2).

Partial variance effect size (*η*^2^) showed large effect size (*η*^2^ = 0.56) and observed power = 1 (α = 0.05) for the factor EVI, large effect size (*η*^2^ = 0.54) and observed power = 1 (α = 0.05) for the factor IVI and large effect size (*η*^2^ = 0.4) and observed power = 0.99 (α = 0.05) for the factor KVI.

### 3.5. SPO_2_ and Heart Rate

Repeated measures analysis ANOVA within subjects for measuring SPO_2_ (final value—intersessions) after 4 weeks between the two groups showed statistically significant results in the means after each session of intervention (F = 5.136, S = 0.001, *p* < 0.05) while no statistically significant differences were observed between the two groups. Furthermore, repeated measures analysis ANOVA within subjects for measuring HR after 4 weeks between the two groups showed statistically significant results in the means (initial and final value) after each session of intervention (HR initial: F = 7.601, S = 0.000, *p* < 0.05, HR final: F = 3.838, S = 0.005, *p* < 0.05), (Table 5).

The means of SPO_2_ (final value) showed an increase of 0.8% in the 1st MI group compared to the 2nd Placebo group, which showed an increase of 0.1% after the six sessions of intervention. The means of HR (initial value) showed a reduction of 1 bpm in the 1st MI group compared to the 2nd Placebo group, which showed a reduction of 8.1 bpm between the six sessions.

The *t*-test for independent samples showed statistically significant differences for the HR (final value) in the first—MI group compared to the second—Placebo group in all six sessions of interventions, (first session: *t* = −2.227, S (two-tailed) = 0.030, *p* < 0.05, second session: *t* = −5.135, S (two-tailed) = 0.001, *p* < 0.05, third session: *t* = −7.107, S (two-tailed) = 0.001, *p* < 0.05, fourth session: *t* = −8.244, S (two-tailed) = 0.001, *p* < 0.05, fifth session: *t* = −7.803, S (two-tailed) = 0.001, *p* < 0.05, sixth session: *t* = −9.144, S (two-tailed) = 0.001, *p* < 0.05), Appendix A. The means of HR (final value) showed a statistically significant reduction of 1.8 bpm in the first—MI group compared to the second—Placebo group, which showed a reduction of 8.2 bpm between the six sessions.

Partial variance effect size (*η*^2^) showed a large effect size (*η*^2^ = 0.33) and observed power = 0.97 (α = 0.05) for the SPO_2_ (final value); also, for the HR (initial value) showed a large effect size (*η*^2^ = 0.42) and observed power = 0.99 (α = 0.05) and for the HR (final value) it showed large effect size (*η*^2^ = 0.27) and observed power = 0.91 (α = 0.05).

## 4. Discussion

The present study evaluated the effects of MI on balance (static and dynamic) and on the fear of re-injury in professional football players with Grade II ankle sprains. The MI was found to have an effective therapeutic outcome only on static balance, specifically on the right leg in OE conditions and on the responses of the ANS through the HR final value, where the results revealed statistically significant differences between the 1st MI group and the 2nd Placebo group before and after 4 weeks of interventions. MI has been considered an effective adjunct therapeutic modality in sports rehabilitation either for injury management (e.g., pain, muscle strength) or sports performance practices (e.g., injury anxiety and fear of re-injury) [26,28,29,49]. There are a variety of methodological designs and approaches regarding the MI intervention; however, most of them are restricted to the healthy athletic population.

In our study, the results of SLST with the use of the KForce plates system in order to measure CoP revealed statistically significant results within subjects after 4 weeks of interventions between the two groups (MI intervention group and Placebo intervention group). There was a greater improvement in the 1st MI intervention group between the 2 Phases for both legs in the OE condition compared to the 2nd Placebo intervention group. Also, statistically significant differences were found for the right leg in the OE condition in the 1st MI group compared to the 2nd Placebo group. On the contrary, our findings did not reveal statistically significant differences in the CE condition in either leg. This is the first single-blind randomized clinical trial aiming to investigate the effects of MI on SLST in professional football players with Grade II ankle sprains who were in the return to play period of rehabilitation. Furthermore, there is a considerable number of studies that evaluated the effects of MI on static balance in the non-athletic population. However, these studies focused on different subject allocation criteria (e.g., stroke patients), thus presenting variations in MI’s efficacious results [50]. Specifically, Bae et al. [51] investigated the effects of MI on balance in patients with subacute stroke. Their findings revealed that there was a statistically significant improvement in balance measured by the Berg balance scale in the experimental group. Similarly to our methodological design, their study had a duration of 4 weeks as well, but the number and the duration of the MI intervention sessions were 12 in total, and they were also task-dependent based on the population’s pathology.

Additionally, in our study, the results of the YBT revealed statistically significant results within subjects before and after 4 weeks between the two groups for measuring dynamic balance in both legs, while no statistically significant differences were found between the two groups and both legs. However, previous studies investigated the effects of MI in athletes with ankle sprains [28,29,35] and did not include the evaluation of the YBT. Nunes and Noronha [35] aimed to investigate the effects of MI in amateur football players with acute ankle sprains. In their case, dynamic balance was evaluated with the use of the Star Excursion Balance Test and their findings did not reveal statistically significant differences in either leg or in either group (MI group and control group). On the contrary, our findings showed the efficacious effect of MI on dynamic balance in professional football players and on the return to play period of rehabilitation and, therefore, training loads and demands were different.

Previous studies investigated the relationship between psychological symptoms and physical injuries in football players [52,53]. The effectiveness of MI interventions in the field of sports psychology has been extensively investigated in regard to sports performance purposes (e.g., self-confidence) [27,54,55,56]. In our study, statistically significant results ‘within subjects’ were observed on the levels of athletes’ fear of re-injury during the return to sport period, while no statistically significant differences were found between the two groups. The levels of fear of re-injury of the CR-IWQ in both factors (worry due to rehabilitation and worry due to the opponent’s ability) were reduced with the use of MI in addition to the balance training program. A limited number of studies have explored the effects of MI on the psychology of an injured athlete during the return to play period of rehabilitation. Cupal and Brewer [49] investigated the effects of MI on re-injury anxiety in athletes after anterior cruciate ligament reconstruction. Their findings showed a statistically significant difference in the re-injury anxiety levels after the MI intervention between the Relaxation and Guided Imagery group in comparison to the Placebo group (attention, encouragement, and support) and the Control group that only followed their physical therapy program [49].

Additionally, in our study, statistically significant results within subjects who took the VMIQ-2-GR before and after 4 weeks of intervention (6 sessions) showed that professional football players had an improvement in their MI ability in all three MI perspectives. However, no statistically significant differences were found between the two groups. The EVI appeared to have the highest improvement, and the IVI had the lowest score, thus being the preferable MI perspective for professional athletes. These findings are in agreement with the study conducted by Hardy and Callow [57], who investigated the relationship between EVI and IVI on performance in professional athletes. In that study, their results supported the view that IVI was more effective when the athlete was more experienced since it enhanced sports performance.

The ANS correlates with MI through the creation of mental movements and the ability to control the visualization [58]. Cardiovascular and respiratory adaptations are the most common physiological responses of the ANS during sports activities [58,59,60]. Mental rehearsals could reproduce these responses and, therefore, evaluate the effectiveness of MI. Comparatively, Ferreira Dias Kanthack et al. [60] aimed to investigate the effects of MI on breath-hold performance by measuring SPO_2_ and HR in amateur athletes. The results showed that performing the task ‘MI of breathing’ was improved compared to the task ‘MI of breath-hold’ and ‘MI trials’ with higher rates of SPO_2_ and HR. In our study, the rates of SPO_2_ (final value) and HR (initial and final value) showed statistically significant results within subjects before and after 4 weeks between the two groups. Only the HR (final value) showed a statistically significant reduction in the 1st—MI group compared to the 2nd Placebo group in all six sessions of interventions, a finding which was not expected. Therefore, the negative relationship between SPO_2_ and HR could be attributed to the content of MI, which was combined with relaxation techniques.

The present study has important practical applications for various reasons. It is the first study to investigate the effects of MI on static and dynamic balance and on the fear of re-injury in professional football players with Grade II ankle sprains and therefore makes it valuable in terms of research and in the clinical application of MI. Our findings proved the MI intervention an effective therapeutic modality in the sports field for physical therapists, coaches and practitioners who desire to potentially improve the rehabilitation process during the return to play period [61]. The use of MI as an adjunct therapeutic modality in addition to a balance training program, will aid professionals in the field to accelerate sports injury management and sports performance after an initial LAS [62].

### Limitations

Although this single-blind randomized clinical trial showed the statistically significant effect of MI on static balance and on the responses of the ANS through the final value of HR in professional football players with Grade II ankle sprains, the duration of MI intervention (4 weeks) was taken into account as the main limitation of the study. Future research could explore the effects of MI during all stages of rehabilitation (acute stage—return to sport), thus continuously evaluating psychological factors with respect to sports trauma and the fear of re-injury. Another possible limitation of our study is the lack of a follow-up after the athlete’s return to sports activities. Psychological alterations due to training intensity and the stress of competition could alter the levels of fear of re-injury, thus affecting sports performance. In the MI content of our study, we included relaxation instructions in order to prepare the athlete to create mental images more vividly. These instructions could have affected the responses of the ANS. Future studies could apply MI in addition to physiotherapy programs without including relaxation techniques. Furthermore, the lack of a control group executing only a balance training program could have influenced the results of the present study. Also, the lack of sports-specific evaluations during the return to play period (e.g., linear sprints and changes of direction) could have limited the information regarding the efficacious effects of MI on static and dynamic balance and on the fear of re-injury during the return to play period after an acute LAS [63,64].

## 5. Conclusions

The aim of the present study was to investigate the effects of MI on balance and on the fear of re-injury in professional football players with Grade II ankle sprain.

Our findings suggest that MI is an effective adjunct therapeutic modality in addition to conventional physiotherapy, including a balance training program. The statistically significant effects of MI intervention on static balance and on the responses of the ANS, as indicated through the HR, suggest that further research is needed in order to establish imagery treatments as a key element of recovery from sports trauma, in combination with psychophysiological factors.

## Figures and Tables

**Table 1:** Repeated measures analysis ANOVA showed statistically significant results of static balance in the OE condition in both legs (SLST) through the CoP (mm^2^) between the two groups (MI and Placebo). The *t*-test for independent samples showed statistically significant differences in the 1st MI group on static balance in the OE for the right leg.

**Static Balance in the OE Condition of the Left Leg for Measuring CoP (mm^2^)**					
**Groups** **N = 58**	**Μ ± SD (mm^2^)** **Pre**	**Μ ± SD (mm^2^)** **Post**	**Mean Difference (mm^2^)**	**Confidence Interval (CI) 95%**	**Sign**
**1st—ΜΙ (n = 29)**	615.10 ± 190.09	496.57 ± 183.17	101.82	37.47; 166.17	F = 10.049 S * = 0.002 *p* < 0.05
**2nd—Placebo (n = 29)**	628.54 ± 261.48	543.42 ± 232.02			
**Static balance in the OE condition of the right leg for measuring CoP (mm^2^)**					
**Groups** ** N = 58**	**Μ ± SD (mm^2^)** ** Pre**	**Μ ± SD (mm^2^)** ** Post**	**Mean Difference (mm^2^)**	**Confidence Interval (CI) 95%**	**Sign**
**1st—ΜΙ (n = 29)**	447.68 ± 157.10	387.77 ± 176.09	206.5	98.4; 314.6	F = 5.523 S * = 0.022 *p* < 0.05
**2nd—Placebo (n = 29)**	655.52 ± 261.52	592.95 ± 290.15			
***t*-test for independent samples for the static balance in the OE condition of the right leg for measuring CoP (mm^2^)**					
**Groups** ** N= 58**	**Μ ± SD (mm^2^)** ** Post**		**Mean Difference (MD) (mm^2^)**	**Confidence Interval (CI) 95%**	**Significance (2-tailed)**
**1st—ΜΙ (n = 29)**	387.77 ± 176.09		205.17	78.92; 331.43	*t* = 3.255 S * = 0.002 *p* < 0.05
**2nd—Placebo (n = 29)**	592.95 ± 290.15				

* S = significant *p* < 0.05; M: Means; SD: Standard Deviation.

**Table 2:** Repeated measures analysis ANOVA showed statistically significant results for dynamic balance in both legs (YBT) through the composite score (%) between the two groups (MI and Placebo).

**Dynamic Balance of the Left Leg (YBT) through the Composite Score (%)**					
**Groups** **N = 58**	**Μ ± SD (%)** **Pre**	**Μ ± SD (%)** **Post**	**Mean Difference (%)**	**Confidence Interval (CI) 95%**	**Sign**
**1st—ΜΙ (n = 29)**	97.19 ± 8.38	99.39 ± 8.85	2.42	1.74; 6.58	F = 7.622 S * = 0.008 *p* < 0.05
**2nd—Placebo (n = 29)**	95.2 ± 7.74	96.54 ± 8.08			
**Dynamic balance of the right leg (YBT) through the composite score (%)**					
**Groups** ** N = 58**	**Μ ± SD (%)** ** Pre**	**Μ ± SD (%)** ** Post**	**Mean Difference (%)**	**Confidence Interval (CI) 95%**	**Sign**
**1st—ΜΙ (n = 29)**	96.96 ± 8	100.77 ± 8.49	2.65	1.14; 6.46	F = 11.451 S * = 0.001 *p* < 0.05
**2nd—Placebo (n = 29)**	95.45 ± 6.72	96.97 ± 7.99			

* S = significant *p* < 0.05; M: Means; SD: Standard Deviation.

**Table 3:** Repeated measures analysis ANOVA showed statistically significant results for the fear of re-injury through the CR-IWQ.

**Fear of Re-Injury Due to the Rehabilitation through the CR-IWQ (Score)**					
**Groups** **N = 58**	**Μ ± SD (Score)** **Pre**	**Μ ± SD (Score)** **Post**	**Mean Difference (Score)**	**Confidence Interval (CI) 95%**	**Sign**
**1st—ΜΙ (n = 29)**	22.79 ± 12.87	16.24 ± 10.94	2.29	−3.22; 7.81	F = 13.488 S * = 0.001 *p* < 0.05
**2nd—Placebo (n = 29)**	23.00 ± 11.47	20.62 ± 10.41			
**Fear of Re-injury due to the opponent’s ability through the CR-IWQ (score)**					
**Groups** ** N = 58**	**Μ ± SD (score)** **Pre**	**Μ ± SD (score)** ** Post**	**Mean Difference (score)**	**Confidence Interval (CI) 95%**	**Sign**
**1st—ΜΙ (n = 29)**	11.37 ± 4.82	8.72 ± 5.2	0.12	−2.09; 2.33	F = 4.737 S * = 0.034 *p* < 0.05
**2nd—Placebo (n = 29)**	10.17 ± 5.39	10.17 ± 3.57			

* S = significant *p* < 0.05; M: Means; SD: Standard Deviation.

**Table 4:** Repeated measures analysis ANOVA showed statistically significant results of the VMIQ-2-GR in all three factors.

**EVI Perspective of the VMIQ-2-GR (Score)**									
**Groups** **N = 58**	**Μ ± SD (Score)** **1st**	**Μ ± SD (Score)** **2nd**	**Μ ± SD (Score)** **3rd**	**Μ ± SD (Score)** **4th **	**Μ ± SD (Score)** **5th**	**Μ ± SD (Score)** **6th **	**Mean Difference (Score)**	**Confidence Interval (CI) 95%**	**Sign**
**1st—ΜΙ (n = 29)**	28.06 ± 9.80	26.34 ± 8.58	24.24 ± 8.26	23.27 ± 7.67	21.75 ± 7.21	20.06 ± 6.84	2.46	–1.34; 6.26	F = 13.697 S * = 0.000 *p* < 0.05
**2nd—Placebo (n = 29)**	30.51 ± 9.27	30.06 ± 8.57	26.13 ± 7.25	25.13 ± 7.69	23.58 ± 6.97	23.06 ± 7.93			
**IVI perspective of the VMIQ-2-GR (score)**									
**Groups** ** N = 58**	**Μ ± SD (score)** **1st**	**Μ ± SD (score)** **2nd**	**Μ ± SD (score)** **3rd**	**Μ ± SD (score)** **4th**	**Μ ± SD (score)** **5th**	**Μ ± SD (score)** **6th**	**Mean Difference (score)**	**Confidence Interval (CI) 95%**	**Sign**
**1st—ΜΙ (n = 29)**	20.86 ± 7.83	21.58 ± 7.92	20.86 ± 8.10	19.41 ± 7.17	17.96 ± 6.58	16.17 ± 5.73	0.27	–3.19; 3.74	F = 12.191 S * = 0.000 *p* < 0.05
**2nd—Placebo (n = 29)**	23.20 ± 5.39	22.72 ± 7.27	20 ± 6.65	18.72 ± 6.69	17.17 ± 7.14	16.68 ± 7.10			
**KVI perspective of the VMIQ-2GR (score)**									
**Groups** ** N = 58**	**Μ ± SD (score)** **1st**	**Μ ± SD (score)** **2nd**	**Μ ± SD (score)** **3rd**	**Μ ± SD (score)** **4th**	**Μ ± SD (score)** **5th**	**Μ ± SD (score)** **6th**	**Mean Difference (score)**	**Confidence Interval (CI) 95%**	**Sign**
**1st—ΜΙ (n = 29)**	23.34 ± 8.13	21.51 ± 7.27	20.75 ± 7.09	19.24 ± 6.47	18.24 ± 5.71	17.27 ± 5.02	2.48	−0.97; 5.95	F = 6.996 S * = 0.000 *p* < 0.05
**2nd—Placebo (n = 29)**	24.82 ± 8.92	23.41 ± 8.02	22.96 ± 7.76	21.89 ± 7.48	21.82 ± 8.10	20.37 ± 8.29			

* S = significant *p* < 0.05; M: Means; SD: Standard Deviation.

**Table 5:** Repeated measures analysis ANOVA showed statistically significant results for the SPO_2_ (after) HR (before and after 4 weeks) between the two groups (MI and Placebo).

**SPO_2_ (%) Final Value**									
**Groups** **N = 58**	**Μ ± SD (%)** **1st**	**Μ ± SD (%)** **2nd**	**Μ ± SD (%)** **3rd**	**Μ ± SD (%)** **4th**	**Μ ± SD (%)** **5th**	**Μ ± SD (%)** **6th**	**Mean Difference (%)**	**Confidence Interval (CI) 95%**	**Sign**
**1st—ΜΙ (n = 29)**	97.50 ± 0.70	97.55 ± 0.78	97.87 ± 0.73	97.92 ± 0.61	98.17 ± 0.48	98.17 ± 0.62	0.007	–0.19; 0.20	F = 5.136 S * = 0.001 *p* < 0.05
**2nd—Placebo (n = 29)**	97.92 ± 0.64	97.80 ± 0.60	97.86 ± 0.89	97.87 ± 0.72	98.10 ± 0.53	97.68 ± 0.59			
**HR (bpm) initial value**									
**Groups** ** N = 58**	**Μ ± SD (bpm)** **1st**	**Μ ± SD (bpm)** **2nd**	**Μ ± SD (bpm)** **3rd**	**Μ ± SD (bpm)** **4th**	**Μ ± SD (bpm)** **5th**	**Μ ± SD (bpm)** **6th**	**Mean Difference (bpm)**	**Confidence Interval (CI) 95%**	**Sign**
**1st—ΜΙ (n = 29)**	72.93 ± 8.40	74.44 ± 11.15	73.20 ± 9.36	70.17 ± 8.33	71.37 ± 8.24	71.00 ± 6.08	−1.27	−5.51; 2.97	F = 7.601 S * = 0.000 *p* < 0.05
**2nd—Placebo (n = 29)**	75.75 ± 14.30	72.72 ± 11.39	70.27 ± 8.68	68.72 ± 9.27	69.93 ± 7.10	68.10 ± 6.62			
**HR (bpm) final value**									
**Groups** ** N= 58**	**Μ ± SD (bpm)** **1st**	**Μ ± SD (bpm)** **2nd**	**Μ ± SD (bpm)** **3rd**	**Μ ± SD (bpm)** **4th**	**Μ ± SD (bpm)** **5th**	**Μ ± SD (bpm)** **6th**	**Mean Difference (bpm)**	**Confidence Interval (CI) 95%**	**Sign**
**1st—ΜΙ (n = 29)**	78.70 ± 9.04	78.27 ± 9.49	79.95 ± 7.36	78.86 ± 6.20	79.06 ± 7.97	78.07 ± 6.49	−12.99	−16.64; −9.33	F = 3.838 S * = 0.005 *p* < 0.05
**2nd—Placebo (n = 29)**	72.25 ± 12.70	65.83 ± 8.94	65.12 ± 8.49	64.00 ± 7.45	64.38 ± 6.25	63.39 ± 5.70			

* S = significant *p* < 0.05; M: Means; SD: Standard Deviation.

## Data Availability

The data presented in the study are available on request from the corresponding author. The data are not publicly available due to ethical restrictions.

## Appendix A

**Table A1:** Demographic characteristics between the two groups.

Demographic Characteristics Ν = 58	1st ΜΙ Group n = 29	2nd Placebo Group n = 29	Statistical Analysis *p* Value
	Μ ± SD	Μ ± SD	*t*-Test for Independent
**Age**	20.5 ± 3.3	21.2 ± 3.1	ΝS, *p* = 0.37 ^α^, *p* > 0.05
**BMI (kg/m^2^)**	22.8 ± 1.7	21.8 ± 2.1	NS, *p* = 0.05 ^α^, *p* < 0.05
**Yrs of training**	11.0 ± 2.8	11.2 ± 2.6	ΝS, *p* = 0.81 ^α^, *p* > 0.05
**Hrs of training/wk**	11.9 ± 1.6	12.3 ± 1.4	ΝS, *p* = 0.26 ^α^, *p* > 0.05
**Dominant Leg**	**Frequencies %**	**Frequencies %**	**Chi-square**
**Right**	Ν = 25, 86.2%	Ν = 21, 72.4%	ΝS, *p* = 0.19 ^β^, *p* > 0.05
**Left**	Ν = 4, 13.8%	Ν = 8, 27.6%	
**Grade II LAS—Leg**			
**Right**	Ν = 21, 72.4%	Ν = 18,62.1%	ΝS, *p* = 0.40 ^β^, *p* > 0.05
**Left**	Ν = 8, 27.6%	Ν = 11, 37.9%	
**Previous LAS—Leg**			***t*-test for Independent**
**Right**	Ν = 17, 58.6%	Ν = 21, 72.4%	ΝS, *p* = 0.30 ^α^, *p* > 0.05
**Left**	Ν = 6, 20.7%	Ν = 6, 20.7%	
**Both**	Ν = 6, 20.7%	Ν = 2, 6.9%	
**Total number of previous LAS**			**Chi-square for Trends**
**1**	Ν = 17, 58.6%	Ν = 13, 44.8%	ΝS, *p* = 0.35 ^γ^, *p* > 0.05
**2**	Ν = 9, 31.0%	Ν = 12, 41.4%	
**≥3**	Ν = 3, 10.3%	Ν = 4, 13.8%	

NS = no significance, M = Mean, SD = Standard Deviation; ^α^ *t*-test, *p* < 0.05; ^β^ x^2^, *p* < 0.05; ^γ^ x^2^ for trends, *p* < 0.05.

**Table A2:** Statistically significant increase (*t*-test for independent samples) for the HR variable (final value) in the first MI group compared to the second Placebo group—six intervention sessions.

HR (Final Value)—Intersessions				
Groups N = 58	Μ ± SD (bpm) Intersessions	Mean Difference (bpm)	Confidence Interval (CI) 95%	Sign (2-Tailed)
**1st—ΜΙ (n = 29)—1st session**	78.70 ± 9.04	6.44	0.64–12.24	*t* = 2.22 S * = 0.030 *p* < 0.05
**2nd—Placebo (n = 29)—1st session**	72.25 ± 12.70			
**1st—ΜΙ (n = 29)—2nd session**	78.27 ± 9.49	12.43	7.58–17.29	*t* = 5.13 S * = 0.001 *p* < 0.05
**2nd—Placebo (n = 29)—2nd session**	65.83 ± 8.94			
**1st—ΜΙ (n = 29)—3rd session**	79.95 ± 7.36	14.83	10.65–19.01	*t* = 7.10 S * = 0.001 *p* < 0.05
**2nd—Placebo (n = 29)—3rd session**	65.12 ± 8.49			
**1st—ΜΙ (n = 29)—4th session**	78.86 ± 6.20	14.85	11.24–18.46	*t* = 8.24 S * = 0.001 *p* < 0.05
**2nd—Placebo (n = 29)—4th session**	64.00 ± 7.45			
**1st—ΜΙ (n = 29)—5th session**	79.06 ± 7.97	14.68	10.91–18.44	*t* = 7.80 S * = 0.001 *p* < 0.05
**2nd—Placebo (n = 29)—5th session**	64.38 ± 6.25			
**1st—ΜΙ (n = 29)—6th session**	78.07 ± 6.49	14.68	11.46–17.89	*t* = 9.14 S * = 0.001 *p* < 0.05
**2nd—Placebo (n = 29)—6th session**	63.39 ± 5.70			

* S = significant *p* < 0.05.
